# Population Dynamics and Long-Term Trajectory of Dendritic Spines

**DOI:** 10.3389/fnsyn.2018.00025

**Published:** 2018-07-24

**Authors:** Ahmet S. Ozcan, Mehmet S. Ozcan

**Affiliations:** ^1^Machine Intelligence Laboratory, Almaden Research Center, IBM Research, San Jose, CA, United States; ^2^Department of Anesthesiology, University of Oklahoma Health Sciences Center, Oklahoma City, OK, United States

**Keywords:** spine dynamics, plasticity, population, synaptogenesis, dendritic spines, spine growth, pruning, filopodia

## Abstract

Structural plasticity, characterized by the formation and elimination of synapses, plays a big role in learning and long-term memory formation in the brain. The majority of the synapses in the neocortex occur between the axonal boutons and dendritic spines. Therefore, understanding the dynamics of the dendritic spine growth and elimination can provide key insights to the mechanisms of structural plasticity. In addition to learning and memory formation, the connectivity of neural networks affects cognition, perception, and behavior. Unsurprisingly, psychiatric and neurological disorders such as schizophrenia and autism are accompanied by pathological alterations in spine morphology and synapse numbers. Hence, it is vital to develop a model to understand the mechanisms governing dendritic spine dynamics throughout the lifetime. Here, we applied the density dependent Ricker population model to investigate the feasibility of ecological population concepts and mathematical foundations in spine dynamics. The model includes “immigration,” which is based on the filopodia type transient spines, and we show how this effect can potentially stabilize the spine population theoretically. For the long-term dynamics we employed a time dependent carrying capacity based on the brain's metabolic energy allocation. The results show that the mathematical model can explain the spine density fluctuations in the short-term and also account for the long term trends in the developing brain during synaptogenesis and pruning.

## 1. Introduction

Rewiring of biological neural networks via structural plasticity is a fundamental mechanism for continuous learning (Bremner, 2017). Compared to changes only in synaptic strength, structural network changes dramatically increases the memory capacity and flexibility (Chklovskii et al., 2004; Holtmaat and Svoboda, 2009). Most excitatory synapses in the neocortex form between axonal-en passant-boutons and dendritic spines (Bourne and Harris, 2008). Dentritic spines are very active and their populations on dendrites are especially dynamic during activity and learning (Yasumatsu et al., 2008).

**Figure 1:**
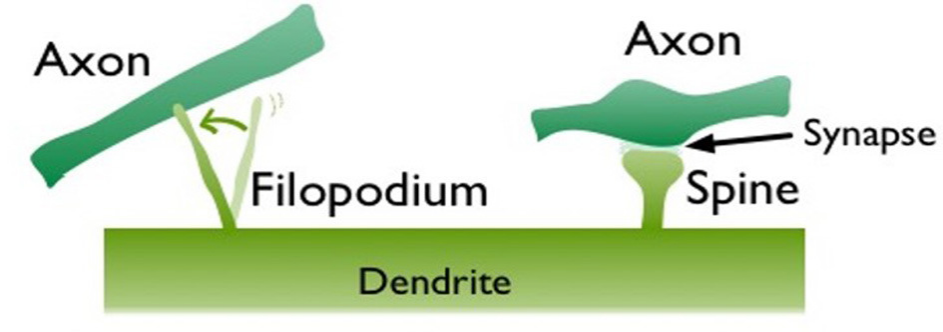
Two different dendritic protrusions in contact with nearby axons. Left is a motile filopodium, which is exploring an axonal partner to form a synapse. On the right, a mushroom shape spine is illustrated, which made a synaptic contact to an axon.

The well-known rules of learning such as the Hebbian learning and spike timing dependent plasticity (STDP) are based on pre- and post-synaptic activity. Synaptic properties can also change spontaneously (i.e., regardless of activity), reinforcing the “dynamic synapse” (Choquet and Triller, 2013) view of neuronal connections. In addition to the spontaneous changes in synaptic-strength, there is also evidence for structural modifications on dendrites independent of activity (Cohen-Cory, 2002). Filopodia, needle-like transient dendritic protrusions, are especially abundant in the neonatal brain (Dailey and Smith, 1996; Ziv and Smith, 1996; Fiala et al., 1998; Jontes and Smith, 2000; Hering and Sheng, 2001; Matus, 2005; Zuo et al., 2005). These protrusions are long,motile, and extremely active structures that can form and retract within minutes. Their morphology is different from the stable mushroom shape spines (Figure 1) and may lack AMPA receptors, which are important for synaptic transmission (Matsuzaki et al., 2001). *In vivo* studies show that filopodia may evolve into stable spines but only a small percentage of them do so (Zuo et al., 2005). Their transient nature and protrusive motility suggest that filopodia provide an alternative synaptogenesis mechanism, which is especially prominent in the developing brain. We previously, postulated that filopodia-type transient protrusions offer a fast plasticity mechanism (Ozcan, 2017). In conjunction with the slower, activity-driven spine growth, this can be a solution for the plasticity-stability dilemma (Mermillod et al., 2013).

In this work, we will focus on the dynamics of dendritic spine populations and develop a mathematical model based on some of the well-known population ecology concepts. The model will cover both the short and the long-term dynamics. By “short-term,” we refer to the local dendritic spine density changes that occur within hours or days, typically in response to a sensory experience or learning task. On the other hand, the global spine density changes that happen in years is referred as “long-term,” which defines the lifetime trajectory of synaptic connectivity.

The dynamics of dendritic spines have been previously studied by Yasumatsu et al. (2008) and a mathematical model has been proposed. In their study, the authors have modeled the spine-head volume as a function time rather than the density of spines on a dendritic branch. The experimental results showed that the volume of individual spines fluctuate stochastically even in the absence of activity. This observation prompted them to adopt the Langevin equation (de Grooth, 1999), which is the fundamental equation for Brownian motion. As a result, their model can predict the spine-head volume fluctuations as a function of time and potentially estimate the average life expectancy of a spine given its volume. However, this approach does not directly provide a model for the spine density (i.e., number of spines per dendritic branch) and it is inherently stochastic since the total spine volume fluctuates which includes spine growth and elimination.

Some other computational approaches (Verzi et al., 2005; Crook et al., 2007) to spine dynamics are based on the standard dimensionless cable equation (Henry et al., 2008), which is used to model the membrane potential change in a passive dendrite. These models include additional equations to represent activity-dependent changes in spine density. In Crook et al, the authors combine their model with one for calcium-mediated spine stem restructuring. These types of approaches focus on the local chemical (e.g., local calcium concentration) and structural characteristics of spines. Also, they are more suitable to study the short-term (1–5,000 ms) dynamics instead of the lifetime trajectory of spine numbers.

A more general rule for spine population changes and cortical rewiring has been proposed by Butz and van Ooyen (Butz et al., 2009; Butz and van Ooyen, 2013). The main idea is the neurons' need for homeostasis in electrical activity, which can guide cortical reorganization. Using a computational model the authors showed that this simple rule can give rise to structural and functional reorganization of neural networks similar to the experimental observations for brain damage.

Contrary to the prior approaches, in this work, we will ignore the stochastic fluctuations of spine volume and focus on the number of spines on a dendritic branch driven by new spine growth and spine elimination. Moreover, we will present a deterministic model, which can be used to predict the spine density trajectory over a long time (potentially the lifetime). Homeostasis is a crucial force in shaping the population in many species, therefore, the rule proposed by Butz and van Ooyen can potentially be incorporated in our model.

## 2. Results

### 2.1. Applying population ecology concepts to dendritic spines

The population dynamics models have mature mathematical foundations and successful applications in a diverse set of practical problems even outside of biology (Neal, 2004; Singh and Uyenoyama, 2004). For example, Gandolfo (2008) applied the predator-prey equations in economics to model resource interactions between industries. Neural network activity has been also modeled (Moreau et al., 1999; Burroni et al., 2017) by population ecology equations to describe the natural oscillations in neuronal networks. In this work, we investigate the feasibility of population ecology modeling for dendritic spine dynamics.

A population, which can be defined as a collection of individuals of a particular species living in a well-defined area, is subject to four factors that can change its size and density. This change can formally be expressed with the following balance equation:

(1) $$\begin{array}{l} \;population\;change=births-deaths+immigration-emigration \end{array}$$

**Figure 2:**
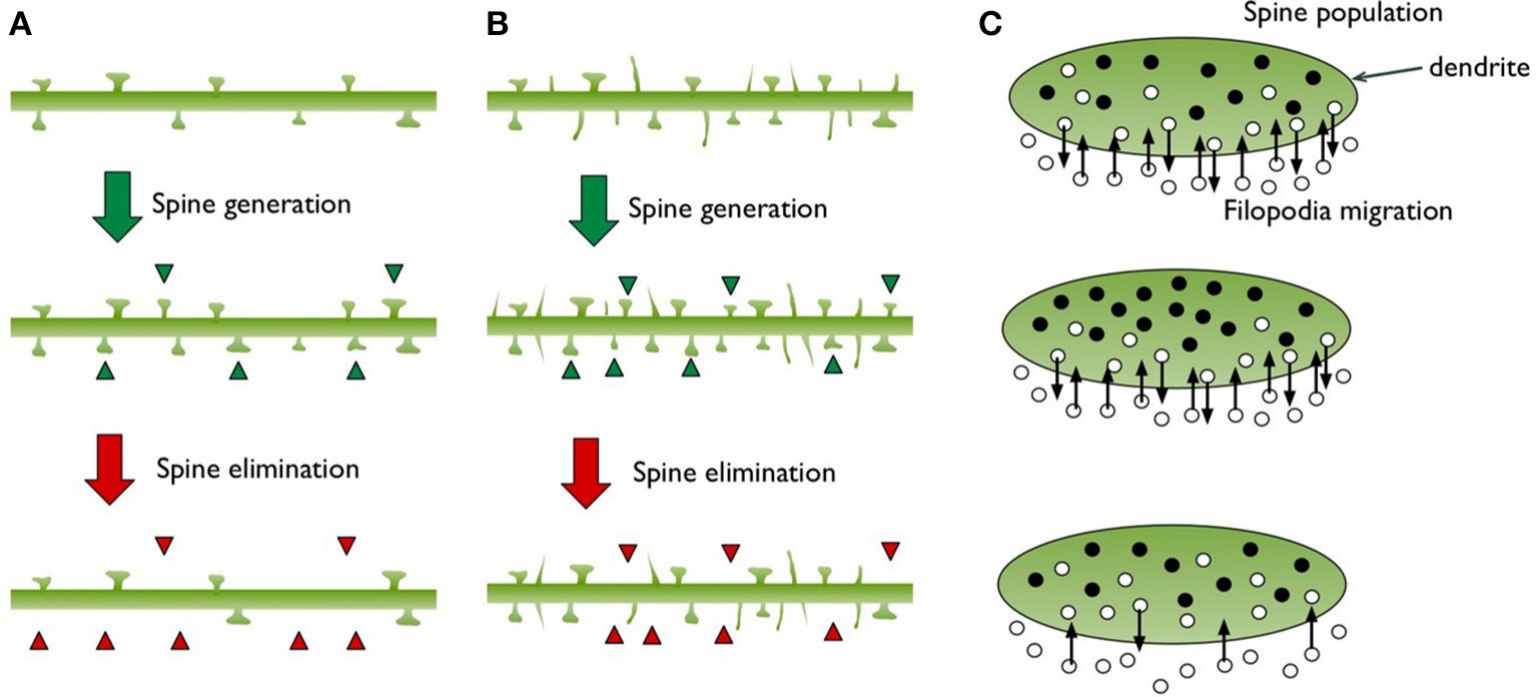
This diagram illustrates the structural changes observed on a dendritic branch during spine generation and elimination. **(A)** Only mushroom shaped spines are depicted, green triangles point to the new spines and red triangles point to the ones that have disappeared. **(B)** Filopodia protrusions are included, which make up roughly 50% of the total population. We illustrate how filopodia can increase the chance of meeting more axons and producing more synapses. **(C)** Based on the spine and filopodia populations in **(B)**, we illustrate the dendrite as a well-defined territory and show spines (filled black circles) as a group residing inside. Filopodia are shown as migrating individuals that go in and out of the territory (small open circles).

We will consider dendritic branches as “well-defined areas” for spine populations, which are typically the subject of experimental studies looking at spine densities. We will model new spine growth as “birth” and spine elimination (pruning) as “death” (Figure 2). The “lifetime” of spines is already a focus of the experimental studies since the balance of plasticity and stability is related to the spine turnover rates.

The concepts of “immigration” and “emigration” in population biology may seem challenging to apply in the dynamics of dentritic spines. In the model we are suggesting, fast appearance and disappearance of filopodia could be viewed as immigration and emigration, respectively (Figure 2C). Our usage of the term “filopodia” here only refers to the spontaneously sprouting thin spines on dendrites. In Figure 2C, the filled circles in the territory (i.e., a dendritic branch) represent spines and the empty circles indicate filopodia, which can enter and exit rapidly as indicated by arrows.

Next we need to consider the other factors for the density dependence of the population growth. The function of synaptic connections on a dendrite is communication with other neurons. Based on the activity dependent learning rules, such as Hebbian and spike timing-dependent plasticity (STDP) (Caporale and Dan, 2008), as the number of synapses per neuron increase the participation of the neuron in a network will grow. Therefore, the probability of new spine growth (i.e., new synaptic connections) will be higher. In the extreme hypothetical case, a neuron with only one synaptic connection would be mostly silent with essentially no chance of participating in neural network, hence, new spine growth (Yuste and Bonhoeffer, 2004) would be highly unlikely.

Populations cannot grow forever and limited resources (e.g., available food) usually limit the growth of the population. In the adult brain, the overall synapse (as well as spine) density is relatively stable over long time scales under normal conditions (Petanjek et al., 2011). In the shorter time scales (hours-days), experimental studies (Xu et al., 2009) show that activity, such as learning new motor skills, causes rapid new spine growth, which is followed by higher rates of spine/synapse elimination. These observations suggest that limiting factors such as the mechanisms suggested for synaptic homeostasis (Pozo and Goda, 2010; Turrigiano, 2012), homeostatis in electrical activity (Butz et al., 2009; Butz and van Ooyen, 2013) or the metabolic energy, eventually stabilize the spine density.

In the light of these considerations, we decided to start with the Ricker population model (Ricker, 1975) which is density dependent. The Ricker model is described by the following equation:

(2) $$\begin{array}{l} \;N_{t+1}=N_{t}e^{r\left(1-\frac{N_{t}}{K}\right)} \end{array}$$

where *N*_*t*_ is the population size at time *t, r* is the growth rate and *K* is the carrying capacity, which represents the maximum population size that can be supported in the environment. The Ricker model assumes that survival depends on the initial cohort size.

**Figure 3:**
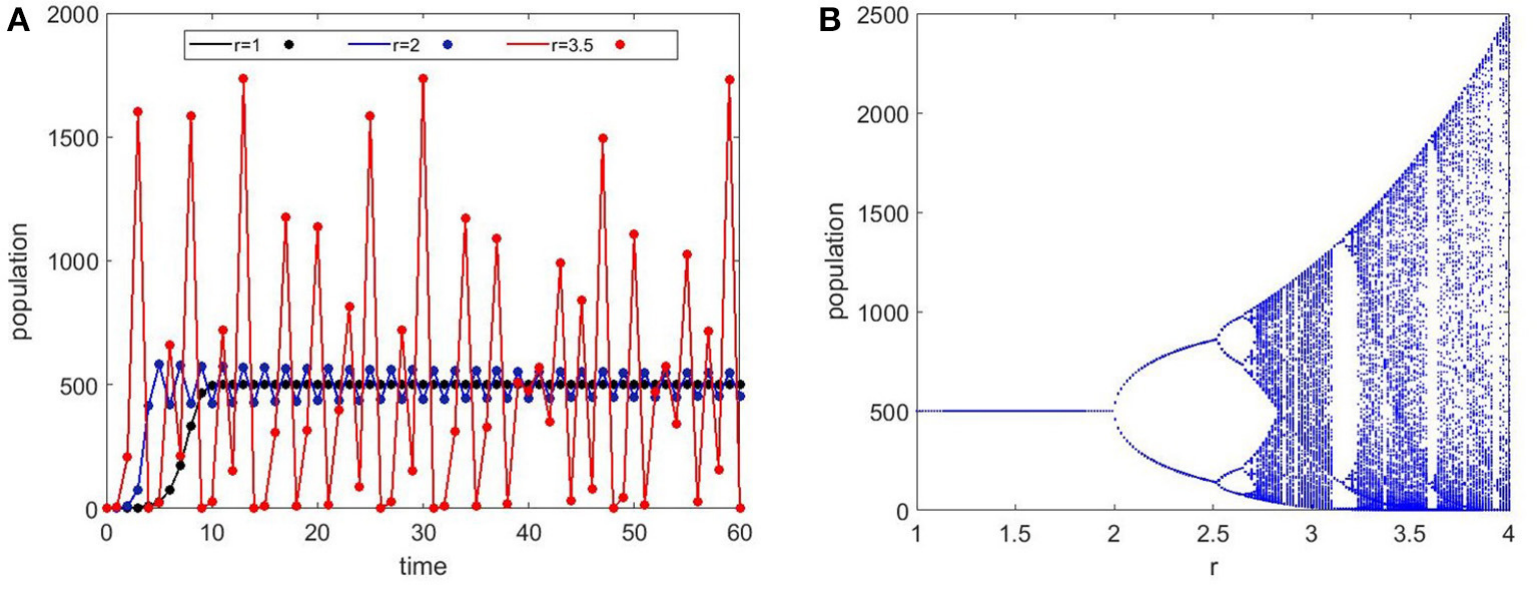
Ricker maps for Equation (1). **(A)** Time series evolution of the Ricker population equation for different values of *r* (small growth rate). The carrying capacity is set constant at 500 for all cases. The black curve with *r* = 1 shows an initial exponential increase that stabilizes at the carrying capacity. At *r* = 2, the population oscillates between two values but stays stable. At *r* = 3.5, we see chaotic oscillations in the population that shows dramatically high numbers as well as extinction. **(B)** The bifurcation map which shows the population as *r* is varied. The period doubling bifurcations lead to chaotic dynamics at about *r* = 2.7.

One of the interesting questions for population dynamics is stability. The population size in the Ricker model is stable for low *r* values (small growth rate) but starts to oscillate as *r* increases and eventually becomes chaotic (Figure 3A). Increasing *r*, first produces period-doubling bifurcations. Then, a sequence of period-halving bifurcations follow, as *r* increases to even larger values. This behavior can be seen in the Ricker bifurcation map (Figure 3B). Chaotic oscillations may look random and can be falsely identified as stochastic behavior. The mathematical use of “chaos” indicates extreme sensitivity to initial conditions and a tendency to fluctuate around a set of values produced by a deterministic equation. There is an on-going debate about whether spine growth dynamics is deterministic or stochastic (Kasai et al., 2010), which is still unanswered. The potential chaotic behavior that emerges from our model is a reminder that seemingly random behavior can in fact be deterministic and should be considered.

Chaotic oscillations of the population density may result in overpopulation or extinction. In the context of spine populations, abnormal levels of spines are directly linked to neuropsychiatric diseases such as autism, schizophrenia and Alzheimer's disease (Penzes et al., 2011; Sekar et al., 2016). Even though experimental evidence for chaotic spine density changes and disease states is not conclusive, it is worthwhile to discuss their potential implications theoretically.

For a population that cycles in time and even displays erratic chaotic behavior, dampening the oscillations is crucial for stability. A number of studies (Ruxton and Rohani, 1998; Stone and Hart, 1999) have indicated that a small number of immigrants entering the population can stabilize the oscillations and prevent chaos. This effect is attributed to creating a “floor,” below which the population cannot fall (Stone and Hart, 1999).

In order to introduce immigration in the Ricker model we can modify Equation (2) by adding a constant parameter, *f* (representing filopodia in our case) which was also shown by Stone and Hart (1999):

(3) $$\begin{array}{l} \;N_{t+1}=N_{t}e^{r\left(1-\frac{N_{t}}{K}\right)}+f \end{array}$$

**Figure 4:**
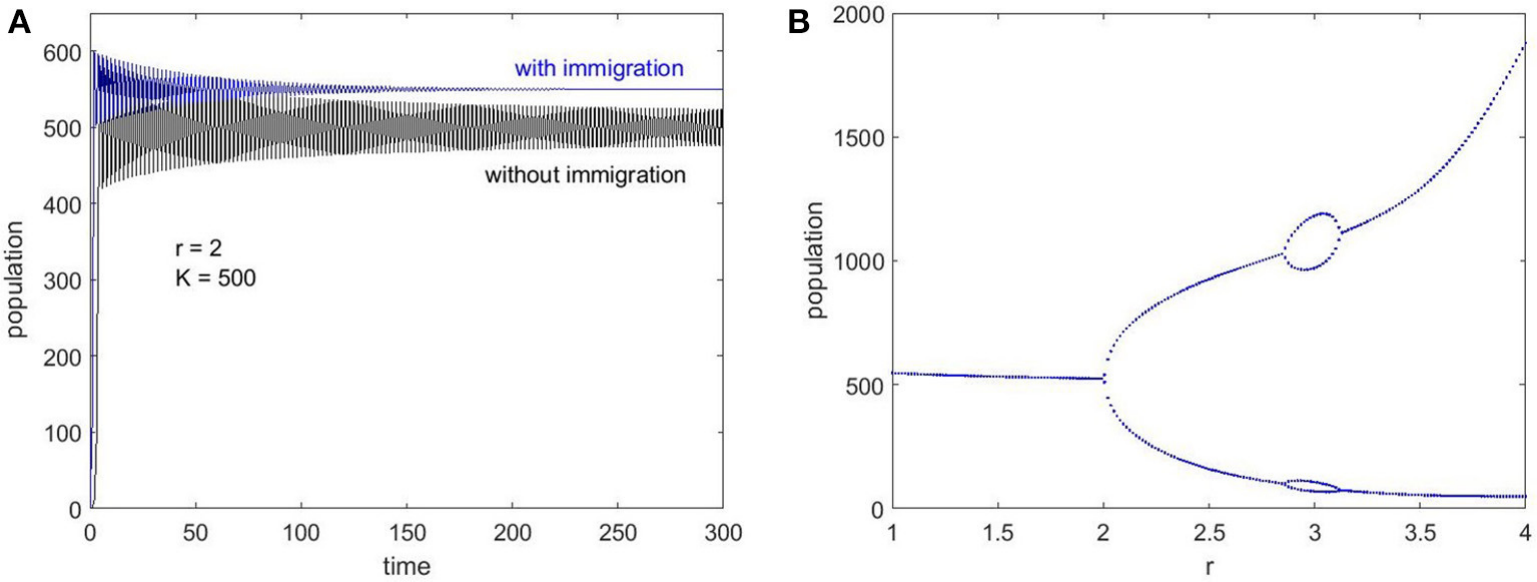
Ricker maps to illustrate the effect of immigration. **(A)** Time series evolution for *f* = 0 (no immigration) and *f* = 100 (with immigration), which demonstrates the stabilization after a return time when there is non-zero immigration. **(B)** Bifurcation map with *f* = 50 shows the absence of chaos, even though there are bifurcations into 2 and 4 point limit cycles as *r* increases.

Plotting the population change as a function of time for *f* = 0 (no immigration) and *f* = 100 (with immigration) shows how immigration affects population dynamics and demonstrate that the oscillations die out in the long term (Figure 4A). Also, the Ricker bifurcation map can be re-plotted with a small amount of immigration to illustrate the absence of chaotic behavior, which is shown in Figure 4B.

A key part of our hypothesis is based upon the observation that there are “activity dependent” and “spontaneous” (i.e., activity independent) spine growth. Similarly, others have referred to “learning spines” and “memory spines” which correspond to thin and mushroom shape spine morphologies respectively (Bourne and Harris, 2008). As mentioned earlier, in this work, we refer to filopodia as spontaneously growing thin spines which are not density dependent. As a biological mechanism, the idea of modeling filopodia as immigrants might be speculative, but mathematically it is justifiable. Previously, it was shown that the chaotic behavior in population density can be suppressed by a non-zero population floor. This population floor can be a different biological mechanism unrelated to filopodia. For example a small percentage of spines that are invulnerable to the density dependent mechanisms can provide stability.

### 2.2. Long-term spine density trajectory

So far we have focused on the short-term and local dynamics of spine populations, which is typically the subject of *in vivo* experimental studies. The global and long-term trajectory of dendritic spine density in the cortex undergoes complex changes during development (Petanjek et al., 2011) and is crucial to understand. Currently our view of the spine density trajectory comes from sampling from different individuals at various stages of the brain development and in specific brain areas (Huttenlocher, 1979, 1990; Petanjek et al., 2011). Some of the most comprehensive experimental studies looking at spine density changes in the neocortex were performed on macaque monkeys (Elston et al., 2009) and chimpanzees (Bianchi et al., 2013). One of the most interesting conclusions of these reports were the difference in macaque monkey and chimpanzees in terms of the cohesion of the spine density trajectory across cortical regions. In the macaque brain, synaptogenesis, pruning and maturation stages seems to be synchronous across different regions (specifically the primary cortex and the prefrontal cortex). However, in the chimpanzee brain, the synaptogenesis period is lengthy and the prefrontal cortex shows an even more prolonged period relative to the other sensorimotor cortical areas. The human brain development shows a similar shift in the prefrontal cortex maturation as observed in the chimpanzee brain (Petanjek et al., 2011). Many psychiatric disorders appear during this critical pruning period, therefore, one of the goals of our model is to look for general principles that dictate these trends.

The statistics of the data in most of the above mentioned studies are not sufficient to estimate the functional form of the spine density evolution. To our knowledge, only Petanjek et al. (2011) have fitted the spine density evolution as a function of time (using a double exponential function) since they collected a very large number of data. They do not provide any explanation for the choice of the double exponential form and they do not provide any conclusions based on the function. We can only speculate that perhaps it was a choice given the wide use of this function for fitting excitatory postsynaptic potential (EPSP) data and the spine density trajectory resembles an EPSP pulse.

The generally accepted view of the spine density evolution includes a sharp increase in the spinogenesis rate during the early years and then a pruning period (Bianchi et al., 2013), which is followed by a relatively stable density during adulthood. The model and main ideas we have introduced in the previous section should apply globally (e.g., the whole cortex or a specific cortical area such as the prefrontal cortex) and at longer time-scales (i.e., years), however, the parameters will be very different because of the sparse activity in the brain. For example, a high spine birth rate locally, which may result in chaotic oscillations in the population will not be observed in the global spine density dynamics, which shows the average trend. Therefore, in this section we will switch gears and think large-scale areas and long-term spine dynamics.

In the Ricker growth equation we described above (Equations 2, 3), the carrying density was assumed to be constant. However, it is reasonable to consider a dynamic time-dependent carrying capacity for the spine population that depends on time. The most obvious parameter that we can relate to spine carrying capacity would be the available metabolic energy. Most of the energy consumed by the brain is due to spiking of neurons for communication (Attwell and Laughlin, 2001; Lennie, 2003; Harris et al., 2012). According to the recent studies on metabolic costs during human brain development, brain glucose uptake reaches adult levels by 2 years old and peaks at around 5 years old and decreases to adult levels later on (Kuzawa et al., 2014), which indicates a similar trajectory to the spine density evolution that we have discussed earlier. These results clearly support the correlation between metabolic energy demand and spine density.

**Figure 5:**
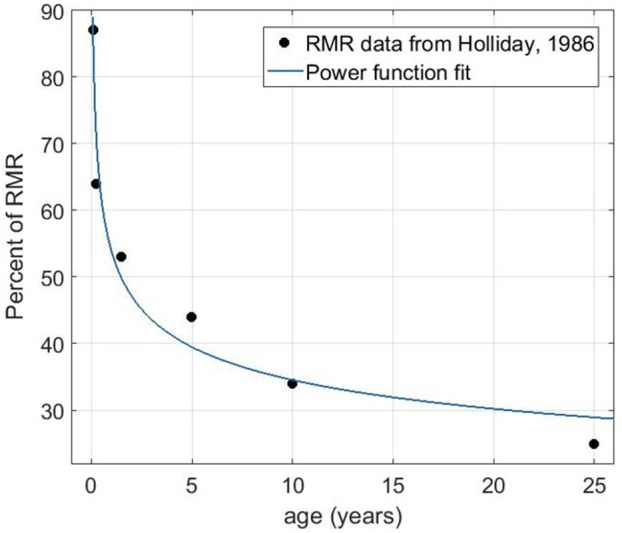
The percentage of resting metabolic rate (RMR) allocated to the brain as a function of age (data from Holliday, 1986) and fit to the data using the power function *f*(*x*)=a*x^*b*^. The fitting gives *a* = 53.88 and *b* = −0.193 with the R-square value of 0.96.

Previous estimates of the percentage of resting metabolic rate (RMR) allocated to the brain per weight suggested a constant decreasing rate from infancy to adulthood (Holliday, 1986) roughly from 85 to 20% as we show in Figure 5 (data obtained from Holliday, 1986 and replotted as a function of age). We used a power function to fit the data points as a function of age, which shows a negative exponent of approximately 0.2. The more complex behavior of brain glucose uptake as reported by Kuzawa et al. (2014), was based on PET and MRI data, and therefore, reflects the actual use or demand of glucose, rather than a maximum budget. The concept of carrying capacity is in the population dynamics is related to the available energy that can sustain the maximum population size. Hence, the monotonously decaying estimate of energy demand per brain weight is still a reasonable assumption to use in our model.

Based on the fit from the RMR percentage by age, we used the same exponent (i.e., −0.2) for the carrying capacity function in our model, which decays by a power law as a function of time. To highlight the time dependence of K, Equation (1) will be modified as:

(4) $$\begin{array}{l} \;N_{t+1}=N_{t}e^{r\left(1-\frac{N_{t}}{K\left(t\right)}\right)} \end{array}$$

where, $K=K_{0}t^{-a}$, *K*_0_ is the initial carrying capacity and *a* is a constant.

**Figure 6:**
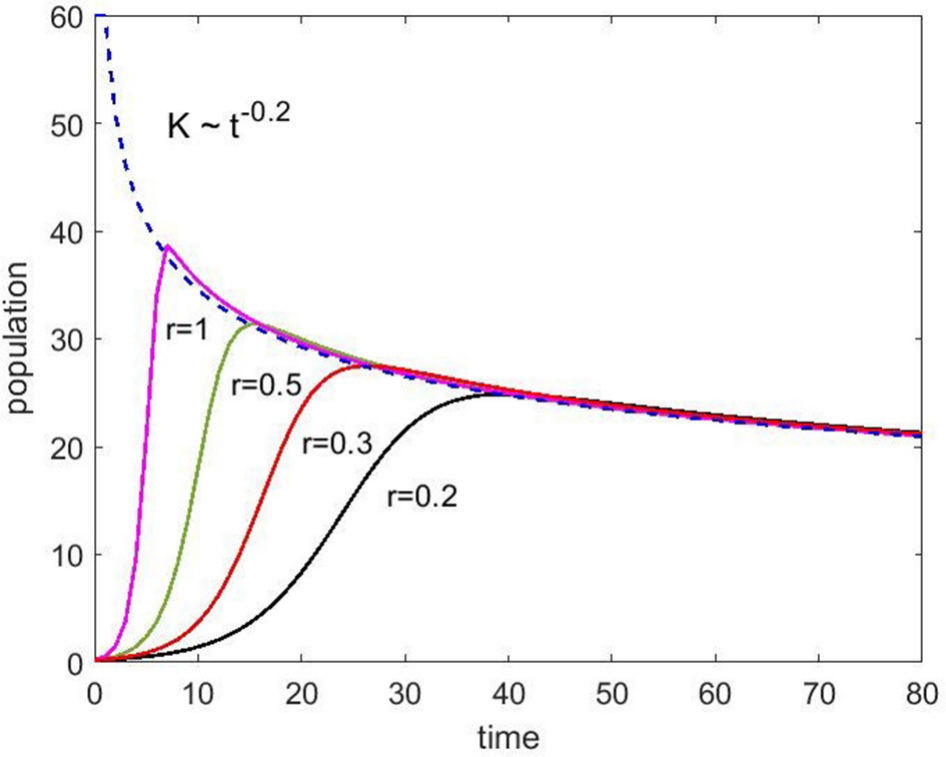
Population change as a function of time without immigration (Equation 4) for various growth rates (*r*) ranging from 0.2 to 1 as indicated. The carrying capacity starts at 60 and decays with the exponent 0.2.

Using Equation (4) (no immigration), we have simulated the population change for different growth rates, r, to show how the time dependent carrying capacity affects population growth (Figure 6). As expected, instead of the saturation at a constant capacity, the population starts to decay after a small overshoot. The numbers on the axes for population and time should be taken as arbitrary but one can get a feel for how the spine density would evolve as a function of years.

Next, we have included the immigration effect to see how it would change the population dynamics in the presence of a time-dependent carrying capacity. Biological data show that filopodia type protrusions are abundant in the post-natal period but decreases quickly during the brain development. For example studies on mice by Zuo et al. (2005) show that more than 50% dendritic protrusions are filopodia in the first two weeks after birth and percentages decay to 10% or below in the adult tissues. Therefore, we chose to start with an initial filopodia number that is 50% of the initial carrying capacity and employed a decaying power function. Equation (3) is modified as:

(5) $$\begin{array}{l} \;N_{t+1}=N_{t}e^{r\left(1-\frac{N_{t}}{K\left(t\right)}\right)}+f\left(t\right) \end{array}$$

where,$f=f_{0}t^{-b}$,*f*_0_ is the initial filopodia density and *b* is a constant. Similar to Equation (4), $K=K_{0}t^{-a}$, *K*_0_ is the initial carrying capacity and *a* is a constant.

**Figure 7:**
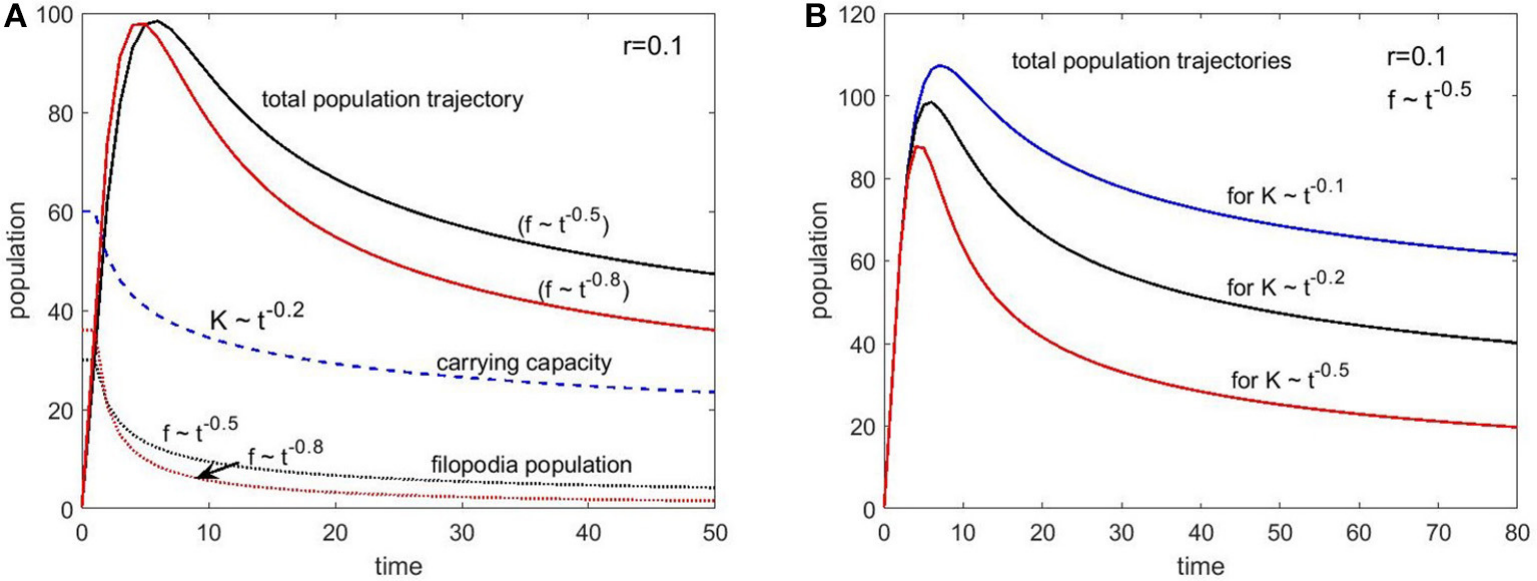
Population change as a function of time with immigration (filopodia) (Equation 5) for different set of parameters. **(A)** The carrying capacity decay is set with 0.2 exponent and two different filopodia population decay conditions are simulated with the exponents 0.5 and 0.8. **(B)** The filopodia decay is kept constant (exponent is 0.5) and three different carrying capacity decay exponents are simulated (0.1, 0.2, and 0.5). The growth rate was the same (*r* = 0.1) for all conditions.

Figure 7A shows two sets of plots for two different filopodia (immigration) decay conditions set by the coefficient b: 0.5 and 0.8. Since the filopodia provide a baseline spine population, this time we see a significant overshoot in the total population compared to the carrying capacity. However, after reaching a certain size, the population starts to decline following the carrying capacity trend. The faster decaying filopodia condition (*b* = 0.8) caused a steeper “pruning” trajectory as expected.

Since our choice for the carrying capacity exponent is only speculative, we explored several values of a to see the impact. Figure 7B shows the population trajectories for these three values of the exponent centered on our initial choice of *a* = 0.2. For all three curves we kept the growth rate and immigration conditions constant (*r* = 0.1 and *b* = 0.5). Biological data on spine numbers from animal and human studies suggest that, from the peak of the synaptogenesis to the stable adult levels we should expect 40–50% decay after the pruning period for healthy individuals. This is what we roughly see in the curve with our chosen exponent of 0.2. This might be a coincidence given all the assumptions made and the simplicity of the model but nevertheless point to the plausibility of these arguments.

**Figure 8:**
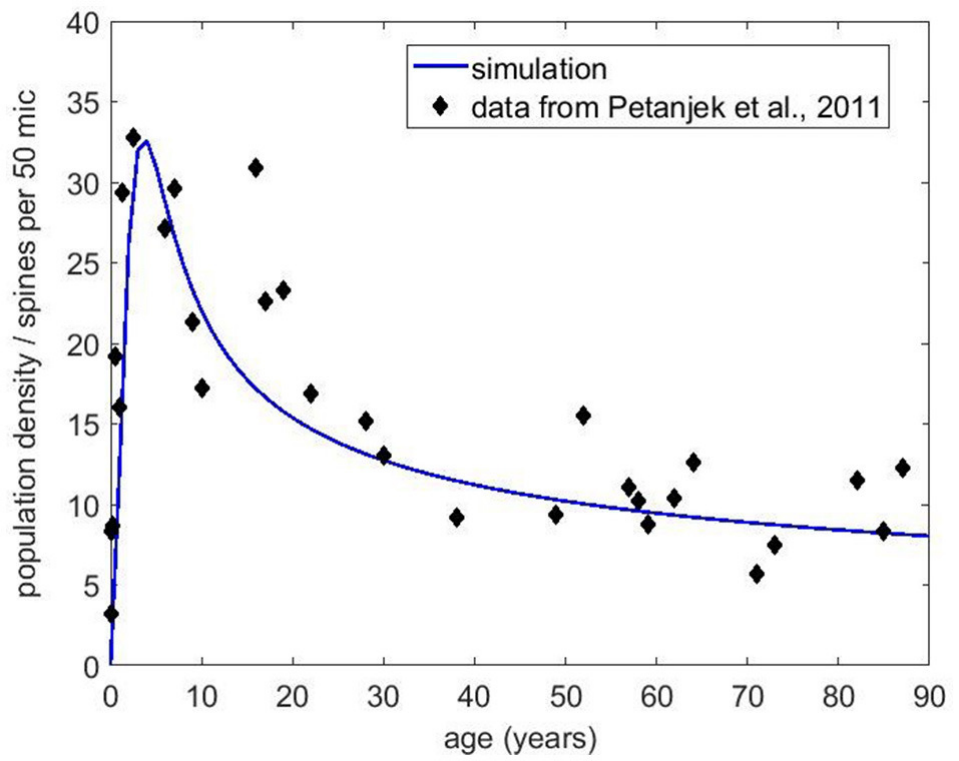
Spine density data obtained from Petanjek et al. (2011) for the basal dendrites in layer III. The blue curve is the simulation results using Equation (5) with *r* = 0.2, initial carrying capacity of 25 and the exponents *a* = 0.3 and *b* = 0.8 for the carrying capacity and the filopodia decay. Initial filopodia population was set at 50% of the carrying capacity.

**Figure 9:**
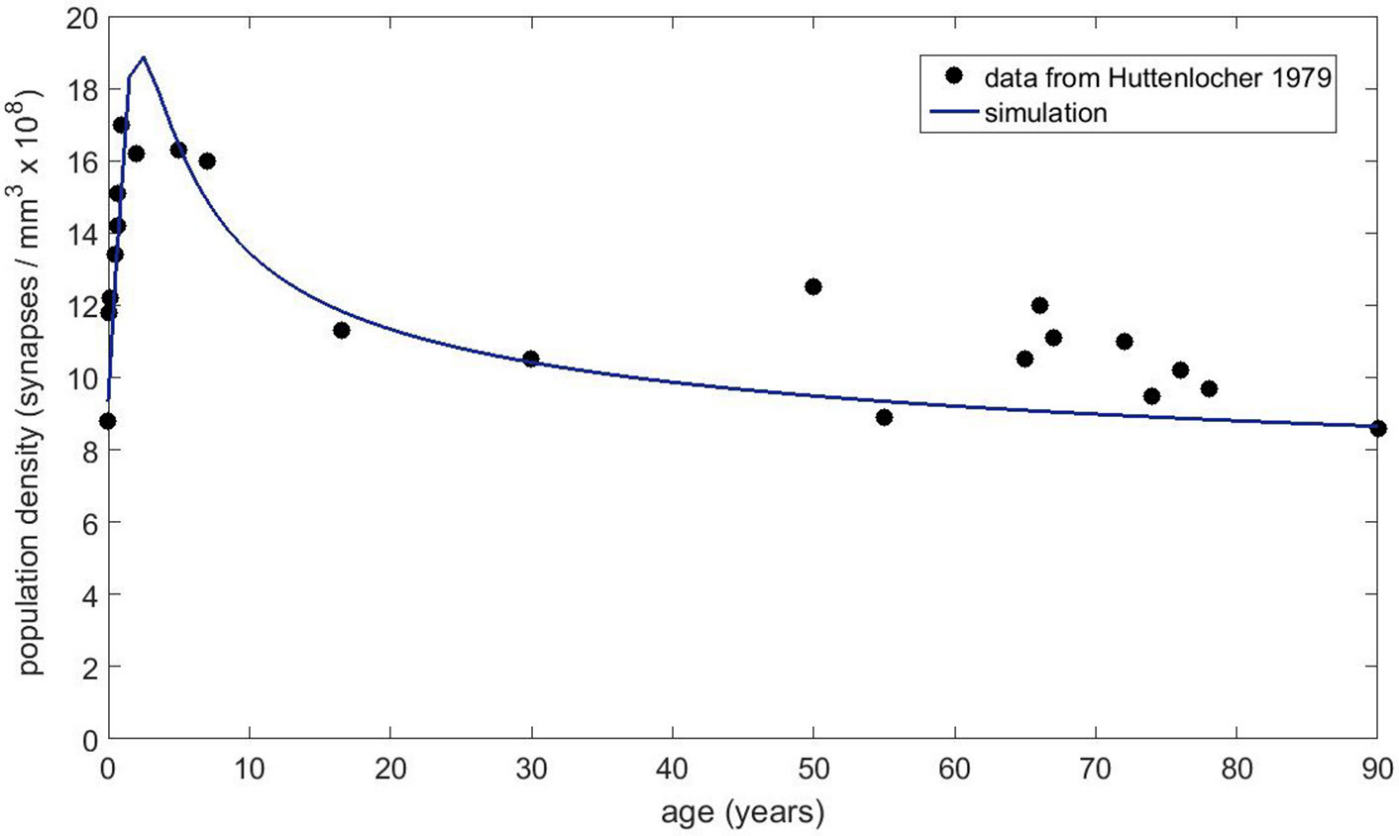
Spine density data obtained from Huttenlocher (1979) for layer 3 of middle frontal gyrus as a function of age.

Next, we obtained the published dendritic spine density data from Petanjek et al. (2011) to compare to our simulations. This dataset is probably the most extensive one for the human brain, as it covers the age range from postnatal to 90 years old with decent statistics. Each data point reflects the mean spine density from many (20–60 samples) dendrites. We chose the basal dendrite dataset from cortical layer III because it contained the most number of measurements and the best statistics. The equation without any immigration factor did not produce satisfactory results (not shown) that can match the data trend for the whole age range, even though it is possible to get good agreement in the first couple decades. However, with the presence of the immigration term, we have found conditions that can fit the data reasonably well. Figure 8 shows the basal dendrite (in cortical layer III) spine density data by age along with our numerical simulation results. In this simulation, the growth rate was set to 0.2, the carrying capacity was 25 and the exponents were *a* = 0.3 and *b* = 0.8 for the carrying capacity and the filopodia decay, respectively. We also compared our model to the older dataset from Huttenlocher (1979), which shows the synapse density in layer 3 of middle frontal gyrus as a function of age. Our simulation results (Figure 9) also show a reasonable agreement with these data over a long period of time.

## 3. Discussion

In the mammalian brain the majority of the excitatory synapses occur on dendritic spines (Yuste and Bonhoeffer, 2004). Therefore, understanding the dynamics of spine generation and elimination is a good measure of synapse dynamics and the evolution of the cortical networks. Even though in vivo experimental capabilities are improving, real-time spine dynamics is extremely difficult to study with good statistics and resolution. On the other hand, the long term changes in spine densities on dendrites are somewhat easier to study since this involves obtaining “snapshots” at different stages of the brain development and one can study a large number of samples (usually from different individuals) in multiple cortical regions (see for example, Bianchi et al., 2013). Nevertheless, the data we have are very sparse, noisy and insufficient to yield a unique mathematical function that can describe the spine density changes, especially for the human brain. As a result, we only have a “putative” model of the spine/synapse density trajectory during the lifetime of mammals. Simply described, this trajectory involves synaptic proliferation after birth that comes to a halt during mid-childhood and subsequent pruning, which lasts until early adulthood. We also know that certain neuropsychiatric diseases, such as schizophrenia and autism, cause a significantly altered trajectory and final spine density (Penzes et al., 2011). Given the experimental difficulties to obtain a more accurate picture of the spine dynamics, an alternative approach is to start with a mathematical model and test its predictions experimentally.

In order to model the complex spine dynamics, we have proposed to use the framework of population ecology. There are many factors to consider before choosing and building the right model. First of all, we made the assumption that the dendritic spine population will be density dependent. We justify this based on two observations: 1-Spine density affects connectivity of the neuron and neural activity is the primary factor for the growth of new spines. 2-*In vivo* studies of dendrites show increase in spine numbers during learning and a subsequent phase of elimination (Penzes et al., 2011), which suggests some pressure to stabilize the spine density. An additional observation is the high rate of “juvenile” mortality (i.e., most of the newly formed spines do not survive and only a small percentage becomes stable), which could be useful to consider.

Based on these assumptions and observations, we have chosen the Ricker model to apply for modeling dendritic spine population dynamics. Ricker's model is based on a density-dependent discrete equation, which was originally developed for predicting recruitment to fishery stocks (Ricker, 1975). The choice of the exact model or the equation is undoubtedly difficult here since we do not have enough data to fit as mentioned earlier. What we are hoping for is the validity of the general approach and investigating the plausibility of a simple and well know population model to predict spine dynamics.

Even though neurons have thousands of synapses, we know that the number of spines saturate at an optimal level, which may depend on the type, age and the location of the neuron. Therefore, it is natural to consider a regulating factor. The Ricker model includes a carrying capacity parameter, which defines the maximum population level that can be sustained. For the short-term spine dynamics we assumed a constant carrying capacity number, which is typical in most applications of the Ricker model.

It is well known that Ricker's equation is stable for smaller growth rates but the population density starts to oscillate at higher rates, which becomes chaotic after a threshold. If the population is not closed, the influx of a small number of immigrants can stabilize the oscillations and prevent chaos, which was observed in island populations and has been studied in detail mathematically (Stone and Hart, 1999). Our calculations reiterated and confirmed these results, bringing a new perspective for the spine dynamics, even though the presence of chaos in spine dynamics is speculative at this point. Given their unique structural and functional properties, we attempted to model filopodia type protrusions as immigrants, which appear rapidly on dendritic branches and operate at a different time-scale compared to the spines that grow more slowly in the presence of persistent neural activity. Although the immigration-filopodia connection is speculative, there is sufficient experimental support that suggests that filopodia population dynamics (Hering and Sheng, 2001) should be monitored and treated separately from the spine (i.e., mushroom type spines) dynamics.

We first showed the results of our calculations without immigration, which is basically the standard Ricker model. For the long-term dynamics, the carrying capacity is modeled as a decaying power function. We base this choice on the estimates for brain's energy demand per weight and its expected trajectory from infancy to adulthood. Since this decay is very slow, the carrying capacity is assumed to be constant at a certain age when we discuss the short-term dynamics. We would like to stress that the constant decay is the trend we use for the carrying capacity change by age and not the actual energy consumption. The actual energy use, which can be estimated based on the glucose uptake, will be related to the spine density and follow a different trajectory (i.e., the trajectory of the population), as confirmed by the recent work of Kuzawa et al. (2014). The carrying capacity can include other factors in addition to metabolic energy, however, considering the fact that synapses consume the most energy in the brain, it is likely that the detailed homeostatic mechanisms like the synaptic receptor alterations are ultimately determined by energy considerations. Also, there are established links between the disorders of synaptic energetics and neurological diseases (Harris et al., 2012) that support our choice.

Our results with the immigration factor (i.e., the filopodia) is intriguing given the observed impact on the lifetime spine population trajectory. For example, when the filopodia percentage declines faster during the spinogenesis period, the pruning trend is steeper and shifts the population curve, even though the growth rate and the carrying capacity were kept the same. This could potentially explain how spine density abnormalities can occur and cause psychiatric disorders. The root cause for the difference in the filopodia exponent could be genetic but nevertheless, the model provides key insight on the mechanisms that control the spine density. The observed prolonged pruning and maturation of the human prefrontal cortex can also be reproduced by this model by adjusting the filopodia parameter. Dumitriu et al. (2010) studied the impact of aging on spine densities in the prefrontal cortex and reported that only the filopodia type protrusions decline and mushroom shape spine density large remains constant. This result supports our model and provides evidence for the different time dependencies for spine and filopodia populations.

The ideas presented here are clearly speculative and need to be tested with experimental studies where filopodia and spine dynamics are monitored carefully both at short and long time scales. However, the simplicity of the model which is based only on several parameters, is attractive to consider. If our intuitions regarding the filopodia dynamics and the carrying capacity are correct, controlling the filopodia density and metabolic energy resources can be crucial for treating or preventing neuropsychiatric disorders.

## 4. Conclusions

Experimental evidence shows that synaptic events are highly dynamic from local alterations to larger topographical changes. One of the key mechanisms that underlies these changes is dendritic spine activity. We have applied a variation of the deterministic density dependent Ricker population equation to model the dendritic spine dynamics in the neocortex and show that population ecology concepts can provide new insights for short-term spine activity, as well as for the lifetime trajectory of dendritic spines. Some of the testable predictions that emerged from this study are:

1. In the short-term, high rates of spine generation can cause chaotic oscillations that can lead to over-population or extinction locally.
2. Small amounts of “immigration” (or a constant stable spine population) can avoid chaos and stabilize the population.
3. The overall lifetime trajectory of the dendritic spine density can be controlled mainly with three parameters: (i) average spine generation rate, (ii) time-dependent carrying capacity, and (iii) time-dependent filopodia density.

We proposed the use of available metabolic energy supply to model the carrying capacity but one can consider other regulatory mechanisms such as homeostasis in electrical activity and develop the model further.

Finally, we hope that this study will spark interactions between experts in ecology and neuroscience, which can ultimately lead to more accurate models and guide future experimental studies.

## Author contributions

AO conceived the theoretical ideas in this work, developed the mathematical model, ran the simulations and took the lead in writing the manuscript. MO reviewed, provided critical feedback and contributed to the writing of the manuscript.

### Conflict of interest statement

AO was employed by the IBM Corporation and this study was fully funded by IBM Research Almaden. The remaining author declares that the research was conducted in the absence of any commercial or financial relationships that could be construed as a potential conflict of interest.
